# The impact and process of a community-led intervention on reducing environmental inequalities related to physical activity and healthy eating - a pilot study

**DOI:** 10.1186/1471-2458-11-697

**Published:** 2011-09-12

**Authors:** Rachel C Davey, Gemma L Hurst, Graham R Smith, Sarah C Grogan, Judy Kurth

**Affiliations:** 1 Centre for Research & Action in Public Health, Faculty of Health, University of Canberra, University Drive, Canberra, Australia; 2 Centre for Sport, Health & Exercise Research, Staffordshire University, Leek Road, Stoke-on-Trent, UK; 3 Institute for Environment, Sustainability & Regeneration, Staffordshire University, College Road, Stoke-on-Trent, UK; 4 Centre for Health Psychology, Staffordshire University, College Road, Stoke-on-Trent, UK; 5 Stoke on Trent Primary Care Trust, Civic Centre, Glebe Street, Stoke-on-Trent, UK

## Abstract

**Background:**

There is growing recognition that a sedentary lifestyle is being driven, at least in part, by environmental factors that affect individuals' physical activity choices and health behaviours. In other words, the environments in which we live, and with which we interact, have become ones that encourage lifestyle choices that decrease physical activity and encourage over-consumption of foods. However, evidence from community-led interventions to change local neighbourhood environments to support physical activity and healthy eating is lacking. This article summarises the research protocol developed to evaluate a community-led intervention *"My Health Matters" *aimed at reducing health inequalities relating to increasing physical activity and healthy eating as defined by community members *themselves*.

**Methods/Design:**

This study includes three of the most deprived electoral wards in Stoke-on-Trent. In each of these areas, environmental factors including proximity of physical activity spaces, greenspace and leisure facilities, neighbourhood connectivity and walkability, land-use-mix and population density, traffic, safety and crime, and food outlets will be mapped using Geographical Information Systems (GIS). A community postal survey of randomly selected addresses assessing environmental characteristics relating to physical activity, perceived health status, social capital, fruit and vegetable consumption and levels of physical activity will be undertaken (baseline and at 2 year follow-up). Based on baseline findings an intervention will be designed and implemented over a 2 year period that includes the following; use of community participatory research to build effective community partnerships; use of partnership consensus to identify, prioritise and design intervention(s) related to specific health disparities; recruitment of local residents to help with the delivery and sustainability of target intervention(s); and the development of local systems for ongoing monitoring and evaluation of the intervention(s).

**Discussion:**

A community-led and multidisciplinary approach to modifying environmental factors that support and reinforce healthful behaviours may be more successful than focusing on individual behaviour change as this approach does not exclusively rely upon individual will and capacity.

Study findings will be collated in 2012 and, if successful in improving levels of physical activity and healthy eating, will help to inform the design of a larger area-based, cluster randomized controlled trial to determine effectiveness.

## Background

There is growing recognition that the rising incidence of obesity is being driven by environmental factors that affect individuals' physical activity and dietary choices. The environments and neighbourhoods in which we live, and with which we interact, have become ones that, in the main, encourage lifestyle choices that decrease physical activity and promote overconsumption of foods. Previous research has identified broad features of the environment and neighbourhood that are likely to affect health outcomes. These include physical features of the environment (e.g. urban form [1], access to greenspace areas for play and active living [2], pedestrian network [3], active transport [4], access to fast food outlets [5]), provision of services (e.g. healthcare, education), socio-cultural/psychosocial (e.g. ethnic make up, level of social capital and community engagement [6]). There is a strong link between the built environment, health outcomes and inequalities. Research in obesity is moving away from individually orientated theories to broader, more environmentally based approaches for understanding and altering the wider environmental determinants of health behaviours [7]. The multiple, dynamic nature of those factors that might influence health are complex, especially for those who live in circumstances marked by social, economic and environmental disadvantages.

Recent debate about social inequalities in health has moved from what could be described as a Universalist approach to one that gives greater consideration to the importance of time and place [7,8]. The connections between social deprivation and ill-health occur in particular localities and in a particular period. Public health strategies designed to enhance health and reduce social inequalities need to be based within particular localities and take cognizance of social and cultural heritage [9]. However, the development of such strategies is complex, requiring both inter-sectoral collaboration and the active participation of local people in their design and implementation. Research in this area to date has been limited, focusing more on identifying the particular determinants of health rather than attempting to design and evaluate particular local solutions. This study is designed to contribute to this limited body of interventionist research.

While there is a huge body of literature that shows wide health inequities between groups or communities based on socio-economic deprivation, and more recently that "places and neighbourhoods" may be the way in which these inequalities are produced or reduced [10-12] there is very little clear evidence as to what types of interventions are most likely to reduce health inequalities. Place-based interventions re-emerged in public health in the 1980s when the Ottawa Charter [13] promoted the strategy of creating supportive environments that facilitate and support engagement with healthier behaviours. This gave rise to the WHO Healthy City movement where emphasis was placed upon reducing health inequalities through policy initiatives for improving the environment through social and economic change. However, difficulties in applying traditional research methods in evaluating these complex interactions has meant that there is little evidence-base of the impact of area-based community-led interventions. The health inequalities caused by this complex, ecological interaction of factors and conditions are not likely to yield to traditional, linear, causal chain, expert-driven approaches to problem solving. Therefore novel methodological and mixed-method approaches are required and as such, we have designed this pilot study to help meet some of these challenges and to inform a larger study, should its findings be positive.

Our approach aims to enhance the health of a community through promoting greater local involvement in community and health decision making to address the health needs and inequities experienced by the community. The establishment of such an approach within any community requires considerable engagement with the community and other agencies. An understanding of the various processes involved in the development and implementation of such a programme is essential if we are to maximize its transferability.

## Methods/Design

The "*My Health Matters*" project will run over 3 years and aims, in collaboration with local stakeholders in the UK health economy (Stoke-on-Trent Primary Care Trust, the Local Strategic Partnership, Stoke-on-Trent City Council, WHO Healthy City, Stoke-on-Trent Board and Third Sector organisations), to promote health in deprived inner city wards of Stoke-on-Trent, UK by undertaking a community-led intervention aimed at reducing health disparities and the associated environmental determinants related to those of the population who are sedentary and overweight/obese. The "*My Health Matters*" project will focus on adults (16+ years), rather than on children; however, families will be involved in the programme where appropriate.

### Study aims

The aim of our proposed study is to determine the efficacy of a community-led intervention in changing environmental determinants of lifestyle health behaviours by targeting barriers relating to increasing physical activity and healthy eating as defined by community members *themselves*. Information on process and effect size will be used to design a larger controlled trial should the findings be positive.

### Study design

Case study pre-post test intervention in three electoral Ward areas of similar socio-economic deprivation [14] in a deprived inner city in the UK with an estimated population of ~10,000 adults.

Targeted communities in these wards will take part in an intensive community-led project, designed to engage its residents in the development and delivery of a range of health related interventions over a period of 2 years. The intervention will:

1. use community participatory research to build an effective community partnership in order to engage community residents, and strengthen community involvement and participation

2. use partnership consensus to identify, prioritise and design intervention(s) related to specific health disparities (and their relevant environmental determinants and mediators)

3. recruit local residents and Third Sector organisations to help with the delivery and sustainability of the target intervention(s)

4. establish local systems for ongoing monitoring and evaluation of the intervention

5. establish a local Public Health Advisory Group comprised of members from cross-government departments (e.g. urban planning, architecture, health, police and transport) to help facilitate policy change

### Research hypothesis

It is predicted that a community-led intervention (working with the community and multiple agencies collectively to achieve beneficial change in a given factor) will increase the proportion of the target population who are physically active (taking part on at least 3 days/week in moderate intensity sport and active leisure) by 10% more (after a two year of intervention).

### Study population

Our study will include targeted lower level Super Output Area (SOAs), the smallest geographical unit for which census information is available in the UK, in three deprived wards in Stoke-on-Trent, UK (Meir, Burslem South and Bentilee) that are similar with regards to socio-economic status (i.e. in the most deprived 40% of the 2007 Index of Deprivation - (IMD)) and are not undergoing major housing regeneration over the next 3 years. A lower level Super Output Area consists of a population size of ~1,500. These spatial units were chosen because they provide an approximate neighbourhood level of analysis due to their population size and are tied to UK Census data.

### Proposed sample size

Sample size calculations for the independent survey sample are based on Cohen's arcsine transformation for estimating differences between proportions [15], where;

$$\begin{array}{c} \varphi =2\;\text{arcsine}\left(\sqrt{\text{p}}\right)\\ \text{h}=\left|\varphi_{1}-\varphi_{2}\right| \end{array}$$

P is the proportion and h is the effect size to be detected. The best available estimate of the population proportion currently active at the recommended level was that taken from Active People Survey 2 [16] (sub-group NS-SEC 6-8 i.e. those most closely matching the demographic profile of the target population for this study), which was 7.5%. Based on our previous research [17], we anticipate being able to achieve a 10% increase in this population proportion i.e. 17.5% after the community-led intervention. Using Cohen's arcsine formula this gives a standardised effect size of 0.273. Assuming a false positive error rate of .05, statistical power of .8 and this standardised effect size, we have calculated a required sample size of 283 participants at follow-up. Further, assuming a survey response rate of 10% (expected to be low for this target population) the issued sample size will be 2830 surveys.

### Study components

The study will be conducted in 4 overlapping phases over a total of 3 years.

### Phase 1: Baseline -mapping of the built environment and community survey using Geographical Information Systems (GIS)

During Phase 1, novel datasets will be assembled that contain data from existing local, regional and national databases together with data from the Regional Library, Public Health Observatory and Ordnance Survey. Within each ward we will map data at the unit of lower layer Super Output Area (LSOA) neighbourhood. LSOAs consist of 4-6 output areas (OAs) which are the building blocks for the 2001 Census. Each SOA has a minimum population of 1,000 and a mean population of ~1,500. SOAs have the following advantages: (a) they are ultimately composed of groups of postcodes and so facilitate better linkage with other datasets that have postcodes, (b) they provide 100% coverage for a range of socio-economic data from the census, (c) the same unit was used for the Index of Multiple Deprivation 2007 and (d) they will be the standard unit for collection of National Statistics and so provide links with other datasets.

For each LSOA population within the wards, we will map a number of key environmental indices that have been identified from the literature to potentially impact on levels of physical activity and eating [18]. These include;

• Population density

• Access to green space

• Access to local services, shops and food retail (including fresh food retail and "fast" foods)

• Access to physical activity facilities (indoor/outdoor)

• Street connectivity

• Land use mix

• Road traffic levels

• Road traffic accidents (including pedestrians and cyclists)

• Crime and anti-social behaviour

All of the above measures will be calculated around every residential address within the study areas. They will be reported within a defined neighbourhood boundary representing a pedestrian catchment area. These neighbourhood boundaries will be created by measuring a 500 m and 1 kilometre walking distance along all roads and pathways from each residential address. A full description of the methodologies, using Geographical Information Systems (GIS) to create these boundaries [19], and the data sets used can be found in a separate technical report [20].

### Community survey

Addresses will be selected from the postcode address file representing all of the targeted streets within each ward included in the study. Postal questionnaires will be sent to a random sample of addresses within the study area for each of the Lower Super Output Areas (LSOA). For each household a random date will be generated and the adult whose birthday falls closest to this date will be asked to complete the questionnaire (intended to select persons within the household with equal probability).

As an incentive, all participants will be entered in to a prize draw for £200 of High Street vouchers if they complete and return the questionnaire.

All respondents at wave 1 would be issued again at wave 2 (at 24 month follow up). This gives sufficient power to detect the expected 10% difference in population proportions. Since only one person is being randomly selected at addresses which have more than one resident aged 16+, at the analysis stage we would apply weights (equal to the inverse probability of selection) to adjust for this.

The self-report questionnaire will include validated and reliable questions on physical activity [21], behavioural intentions and behavioural change [22], perceived health - SF12 [23], perceptions of the local neighbourhood for physical activity (Neighborhood Environment Walkability Scale) [24], social capital [25] and socio-demographic information (gender, age, ethnicity, education level, household tenure). The questionnaire and survey procedures will be fully piloted before the main fieldwork for wave 1 and for any changes or additional questions at wave 2 (at follow up).

In addition to the survey a random sub-sample of 100 adults from each of the three Wards will be asked to wear an accelerometer (GMT1) for 7 consecutive days to measure physical activity levels [26].

Results from the objective GIS mapping and community survey will be used as the basis of community consultation.

### Phase II: Establish working Groups for intervention planning

Each ward of Stoke-on-Trent, UK has an Area Implementation Team (AIT) which is directly aligned to Stoke-on-Trent City Council, Stoke Primary Care Trust, and the Local Strategic Partnership and involves membership from joint services (e.g. Health, Police and Fire Services, private, community and voluntary sector organisations). The aim here is to bring both the target community and a multidisciplinary professional perspective to the process of planning the intervention. Encouraging individuals and groups for whom the intervention is intended will help optimise sustainable consensus and action. This will be achieved through the AIT network by the formation of neighbourhood "Working Groups" with representation from those who are the intended beneficiaries and those implementing the programme taking account of the need for culturally relevant programmes for diverse groups. Community participants are likely to be a mixture of unaffiliated residents, voluntary organizations, staff members from the AIT and leaders from community organizations. It is recognized that over time membership of these groups may change. The task of these groups will be to help determine performance objectives and determinants to develop an overall matrix of change objective grid; this process is described in Phase III. The AIT Network will help with the setting up and coordination of these groups and a dedicated Community Development Worker (CDW) will be allocated to each area. These CDWs will act as agents and catalysts for change and will work across several levels and will be responsible for increasing motivation and demand for health. This will be achieved through an approach which combines community engagement with supportive policy change that focuses on facilitating and creating healthy urban environments (as identified by community members themselves).

### Phase III: Programme development and implementation

**Figure 1:**
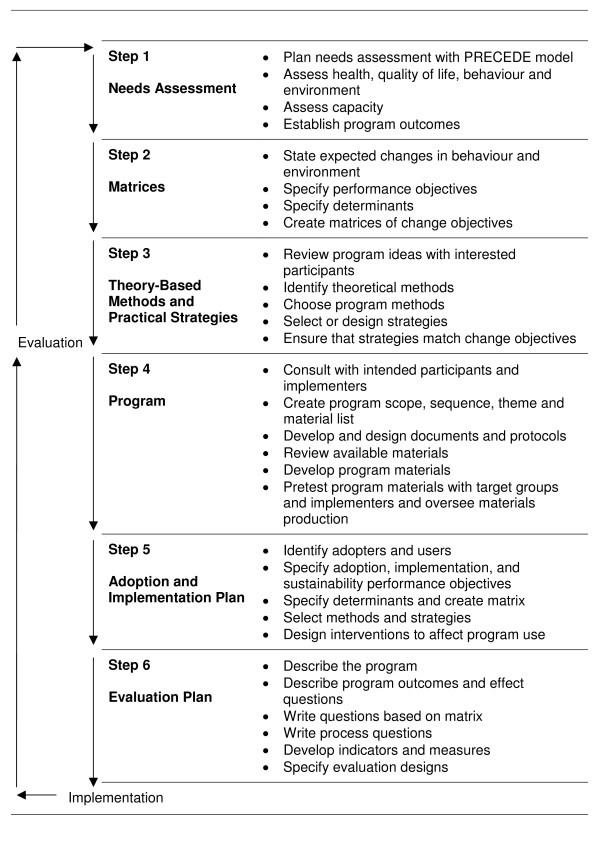
**Intervention Mapping protocol **[28].

The perspective for this part of the programme is the continued use of the ecological framework which recognises that individuals' behaviours are determined by a web of factors that operate at multiple levels [27]. We aim to reduce health inequalities by targeting health-related problems defined by community members themselves. We will use Intervention Mapping (IM) which is a stepwise procedure for the systematic development, implementation and evaluation of health promotion programmes using the social ecological approach (see Figure 1) [28]. In line with ecological models of health behaviour, IM distinguishes between individual and environmental determinants of health behaviour and is an integrative problem driven approach to explore mediators of behaviour change and to identify potential behaviour change strategies. IM is a systematic way to proceed from knowledge about behavioural determinants to specific change goals, and subsequently to intervention methods and strategies, based on the population intervention matrices. Such matrices finally develop into an "intervention map" that makes the translation of objectives for change to actual intervention activities.

This phase provides the foundation of the intervention by specifying who and what will change as a result of the intervention. The aim of this phase is to establish a set of matrices of selected ecological levels (i.e. individual, interpersonal/family, community, organisational, policy through to societal) that combines performance objectives for each level with selected determinants required to produce change objectives. This process will define what needs to be achieved in order to enable changes in behaviour or environmental conditions that will increase physical activity levels. Objectives beyond the individual will include environmental agents selected at each ecological level.

Members of the community organised into "working groups" will be asked to formulate specific programme objectives for each of the key environmental issues related to lifestyle choices, namely (i) individual, (ii) interpersonal/family (iii) social factors, (ii) organisational factors, (iii) policy, and prioritise these based on importance and desirability to change leading to a local health action plan (key activities and actions). For each priority area they will create a matrix of change objectives for each level of intervention planning (individual, interpersonal, organizational, community, and societal) by crossing performance objectives with determinants.

Our approach emphasizes the importance of local relevance and context. Thus the precise health issues and intervention(s) over the 2 years to be addressed cannot be defined *a priori*. The community-led intervention(s) will be facilitated by the CDWs and neighbourhood Area Implementation Teams working in the three intervention Wards.

Programme implementation will be resourced by Stoke-on-Trent Primary Care Trust. We will endeavour to incorporate the intervention programme within mainstream healthcare delivery through local commissioning via the PCT in order to ensure sustainability. We will embed successful elements of the intervention within organisations' routines, and by capacity-building in the recipient communities. As part of the intervention we will use programme "champions" recruited from volunteers in the community and trained to help support and facilitate the adoption of programme objectives.

Throughout the intervention period, we will develop an evaluation framework based on the Logic Model [29] designed to enable partners to monitor programme activities and to assess the extent to which those activities, independently or in combination, influence desired intervention goals. Key stakeholders will be involved to ensure that the results or outcomes are relevant to recipients, funders and policymakers. We will use mixed methods (qualitative and quantitative) to assess the impact of a systematic effort by residents to engage in a collaborative approach leading to culturally sensitive and appropriate action. It will not be feasible to evaluate, with full rigour, all of the individual elements that make up this multi-factorial, multi-agency approach, in which several smaller components are combined to yield an overall effect. Where we are able to do so, we have designed the programme element with sufficient power to detect expected differences in the physical activity levels.

### Phase IV

#### Primary and secondary outcomes

##### Primary outcome measures

The primary outcome measure of effectiveness of this approach will be the change in physical activity in the three areas compared with the other wards in Stoke-on-Trent (using the Active People Survey [16]). The study is designed to detect a 10% increase (intervention areas vs. the rest of Stoke-on-Trent) in the proportion of participants reporting an increase in physical activity, made up of those initially engaging in some activity who increase this to the level recommended to derive health benefit and those initially doing little or no activity who increase to doing some regular activity. Objective measures of physical activity will be used for a random sub-sample of respondents to the community survey using 7-day accelerometry (ActiGraph) as a check on self-reported physical activity measures [26].

##### Secondary Outcome measures

These will include; proportion of the population eating 5 portions of fruit/vegetables a day, perceived health (SF-12), a sub-set of question items used by the Health Survey for England and recommended by the Office for National Statistics (ONS) will be used to assess social capital [25]. Aspects of social capital and social change will be explored in greater depth through focus group discussions on themes related to; social participation, civic partnership, social networks, reciprocity/trust and perception of local neighbourhood environment. We will triangulate qualitative findings from these focus groups with quantitative survey data.

### Assessment of processes

Qualitative data will be collected at various stages of the project. This will involve individual interviews and group discussions with selected residents. This will assess their perceptions of the programme and their integration of it into their everyday lives. These interviews will be conducted prior to the intervention, at least twice during the intervention, and after the intervention has concluded. The interviews will be tape-recorded and subjected to thematic analysis [30]. In addition, interviews will be conducted with a sample of the various social and health professionals and volunteers involved in the project to identify their perceptions of the programme and the challenges they had encountered in its implementations. Based upon previous comparable work it is intended that for each area 20 residents will be interviewed in one-to-one interviews and there will be 8 group discussions with approximately 6 residents in each group. This is a total of 68 residents together with 10 professionals and volunteers. Written, informed consent will be obtained from study participants. It is intended to supplement the interviews and discussions with substantial ethnographic field notes, narrative diaries (videos and photographs) and other data (e.g. photographs, minutes of meetings, newspaper reports, etc.) collected by the researchers on community meetings and other activities. This broad array of data will be collated and systematised to develop a sophisticated understanding of the processes involved in the development of the intervention [9].

### Data analysis

Chi-squared analysis will be used to test for differences in the distributions of PA categories in the intervention and control areas. Multinomial logistic regression analysis will be used to derive odds ratios for the various physical activity categories between the areas. Qualitative analysis of the interviews and discussion groups will follow the principles of thematic analysis [30]. The interviews will be audio tape-recorded, transcribed and entered onto a database. Then using the qualitative analysis computer package N-Vivo it is planned to conduct a thorough coding of the data followed by identification of basic themes and larger themes.

### Ethical arrangements

Ethical approval has been obtained by the Research Ethics Committee, Staffordshire University.

## Discussion

The need for a community-led collaborative approach to combating social inequalities in health has grown out of the recognition that many of the complex determinants of health lie beyond the control of the individual and even of clinical and public health institutions alone. Social factors have a strong influence on health and longevity. Those in deprived areas often lack access to health services, which reduces their likelihood of having disease detected early, live in areas where there is poor access to fresh affordable food and areas to play and participate in physical activities (green space areas run down, vandalised) and often have a number of lifestyle modifiable risk factors (e.g. smoking, sedentary behaviour, high alcohol intake) that compound these health issues. In addition, those living in poor areas are more likely to suffer from depression, stress and poor mental health [31,32].

There is a need to develop evidence-based practice around health interventions that improve public health of communities. Development of an evaluation framework and dissemination of best practice would add considerably to the current evidence-base and offer possibilities for future service innovation across other NHS PCTs.

## Conclusions

There is a need to develop new approaches to combating health inequalities. A community-led approach is an important component of health improvement policy and practice. However, although these approaches are often described, their impact is seldom evaluated and the process of integrating into local area planning usually ignored. Community participatory research and community-led intervention are grounded in the concepts of "local community" and "local control" and in combination may potentially provide the required social and political combination to address some of the health inequalities related to physical activity.

## Competing interests

The authors declare that they have no competing interests.

## Authors' contributions

The study was originally conceived by RD and GH, SG and JK. GRS provided GIS support to the project. All authors helped draft and revise the manuscript, and read and approved the final version.

## Pre-publication history

The pre-publication history for this paper can be accessed here:

http://www.biomedcentral.com/1471-2458/11/697/prepub
